# Haloperidol changes mRNA expression of a QKI splice variant in human astrocytoma cells

**DOI:** 10.1186/1471-2210-9-6

**Published:** 2009-03-31

**Authors:** Lin Jiang, Peter Saetre, Elena Jazin, Eva Lindholm Carlström

**Affiliations:** 1 Department of Development and Genetics, Uppsala University, Sweden; 2 Department of Clinical Neuroscience, HUBIN project, Karolinska Institute and Hospital, Sweden

## Abstract

**Background:**

*The quaking homolog, KH domain RNA binding (mouse) *(QKI) is a candidate gene for schizophrenia. Disturbed QKI mRNA expression is observed in the prefrontal cortex of patients, and some of these changes correlate to treatment with antipsychotic drugs.

To test if low doses of antipsychotic drugs can modify QKI mRNA expression, human astrocytoma (U343) and oligodendroglioma (HOG) cell lines were treated with five different antipsychotic drugs including Haloperidol, Aripiprazole, Clozapine, Olanzapine and Risperidone. Messenger RNA expression levels of splice variants QKI-5, QKI-6 and QKI-7 were measured by Real-Time PCR.

**Results:**

Haloperidol treatment (0.2 μM) doubled QKI-7 mRNA levels in U343 cells after 6 hours (p-value < 0.02). The effect was dose dependent, and cells treated with ten times higher concentration (2 μM) responded with a five-fold and three-fold increase in QKI-7, 6 and 24 hours after treatment, respectively (p-values < 0.0001).

**Conclusion:**

The results in U343 cells suggest that QKI-7 mRNA expression in human astrocytes is induced by Haloperidol, at concentrations similar to plasma levels relevant to clinical treatment of schizophrenia. The molecular mechanism of action of antipsychotic drugs after binding to receptors is not well known. We hypothesize that QKI regulation is involved in this mechanism.

## Background

QKI was recently proposed as a candidate gene for schizophrenia based on linkage, association and mRNA expression studies [1-3]. The alteration of expression levels involves differential expression of splice variants. An earlier study showed decreased expression levels of QKI splice variant seven (QKI-7) in frontal cortex of schizophrenia patients [2]. QKI controls expression of oligodendrocyte related (OR) genes, as reviewed previously [4], and expression of OR genes has clearly been shown to be altered in schizophrenia [5]. Furthermore, QKI expression was recently shown to be both necessary and sufficient to promote myelination of oligodendrocytes [6]. Astrocytes also play a role in myelination by mediating communication between axons and myelinating glial cells [7] and it has been suggested that these two cell types are involved in the pathophysiology of schizophrenia. [8].

Interestingly, an association between the type of antipsychotic drugs patients received and QKI mRNA expression was shown previously [2]. We hypothesized that antipsychotic medication influences QKI expression. To test this hypothesis we treated human oligodendroglioma (HOG) and astrocytoma (U343) cell lines with five different antipsychotic drugs, at levels similar to clinical plasma concentrations [9-12]. We show that Haloperidol stimulates QKI-7 mRNA expression selectively in U343 cells.

## Results

**Figure 1:**
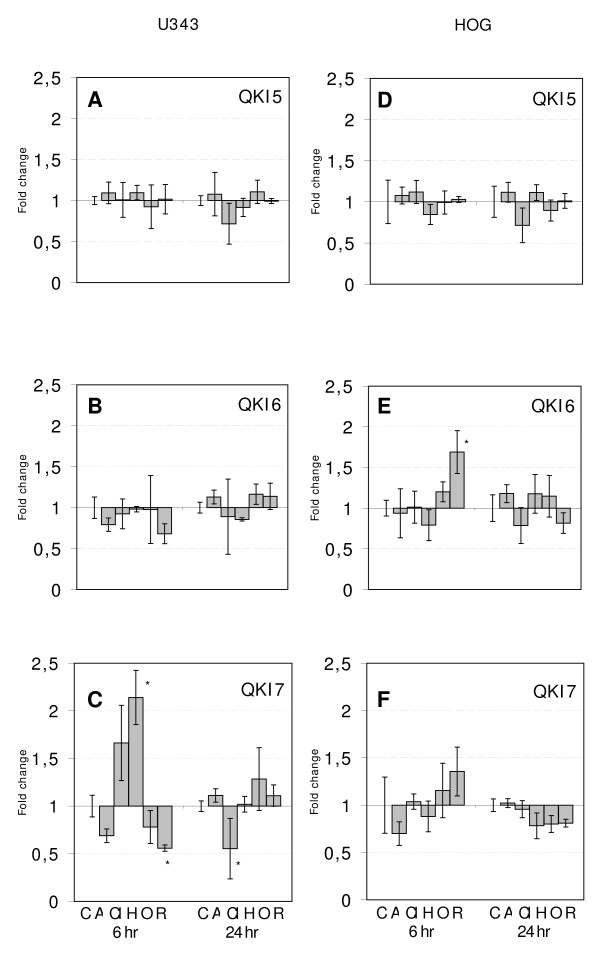
**The graphs show the mean fold change of relative gene expression values of QKI splice variants (QKI5, QKI6 and QKI7) in U343 (1A-C) and HOG cells (1D-F) treated with Aripiprazole (A), Clozapine (Cl), Haloperidol (H), Olanzapine (O) and Risperidone (R)**. The bars for the control samples are indicated with a C. Error bars indicate standard errors. The asterisks indicate results with a p-value of < 0.05.

To test whether antipsychotic drugs affect QKI mRNA expression in U343 and HOG cells, we treated cell cultures with Aripiprazole, Clozapine, Haloperidol, Olanzapine or Risperidone for 6 and 24 hours. In U343 cells, the most significant change in mRNA expression levels was a 2.1-fold increase of QKI-7 (p-value < 0.02) after 6 hour treatment with Haloperidol (Figure 1A–C and Additional file 1, Table 2a). At this concentration the effect appeared to be transient and expression levels in treated cells were similar to those in control cells after 24 hours. A slight decrease in mRNA expression was also detected for QKI-7 when the U343 cell line was treated with Risperidone or Clozapine (Figure 1C and Additional file 1, Table 2a). In HOG cells, we only observed a slight increase of QKI-6 mRNA levels (p-value 0.03) in response to Risperidone (Figure 1D–F and Additional file 1, Table 2b). The increase of QKI-6 in the oligodendroglioma cell line by the atypical antipschotica Risperidone does not reflect previous alterations observed in brains of patients [1-3]. However, it is important to remember that no patients are treated exclusively by Risperidone. Furthermore, changes detected in brain of patients are the result of drug effects on multiple cellular pathways and cannot directly be compared with *in vitro *effects on a single cell line. In any case, our current *in vitro *experiments and the previous analysis of post-mortem brains coincide in the suggestion of alterations in QKI control in both astrocytes and oligodendrocytes after treatment with antypsychotic drugs

**Figure 2:**
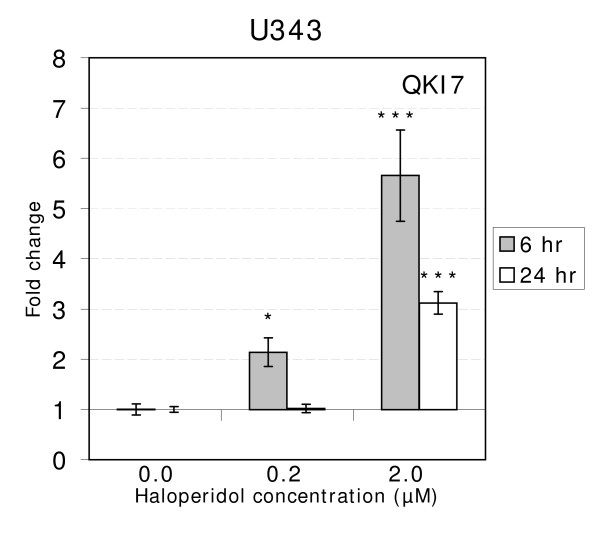
**The graph shows dose-response for Haloperidol concentrations 0 μM, 0.2 μM and 2 μM in treated U343 cells**. The fold-change in quantity is plotted against the drug concentration, for 6 and 24 hours. Error bars indicate standard errors. The asterisks indicate significant results (*** indicate p-values < 0.001).

**Figure 3:**
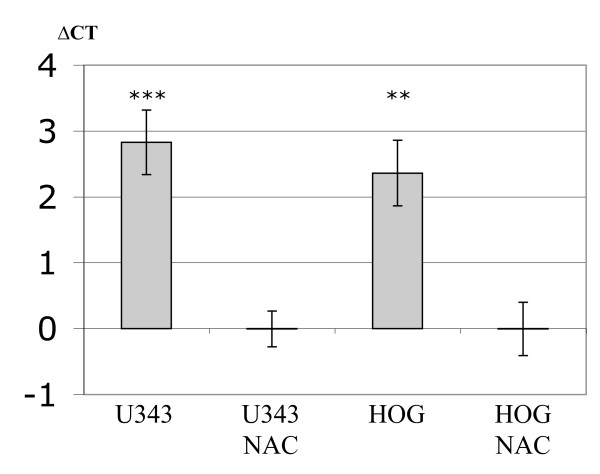
**The graph shows the expression of the dopamine 2 receptor in U343 and HOG cells**. ΔCT are derived by subtracting the CT values obtained from the Negative Amplification Control (samples where no enzyme was added to the reverse transcription) with the CT obtained from the samples. P-value (U343) = 0.0001 and p-value (HOG) = 0.002.

To further evaluate the effect of Haloperidol on QKI-7 mRNA expression, we treated U343 cells with 10 times higher concentration (2 μM) and observed a five-fold increase after 6 hours (p-value < 0.0001) and a three-fold increase after 24 hours (p-value < 0.0001) (Figure 2 and Additional file 1, Table 2c). Antipsychotic drugs are known to bind to dopamine receptor 2 (D2R) and/or 3 (D3R) among other receptors. To evaluate whether dopamine receptors were present in the cells we measured mRNA levels of both receptors in each cell line. We detected low levels of D2R mRNA expression in both U343 and HOG cells (Figure 3). D3R expression was not detected in either of the cell types (data not shown).

## Discussion

A previous study showed that post-mortem samples from schizophrenia patients had different mRNA levels of QKI splice variants depending on the medication received by the patients [2]. However, it was not evaluated whether QKI alterations were due to a direct or indirect effect on medication, and what cell type(s) were involved. In this study, we show that treatment of a human astrocytoma cell line with the typical antipsychotic agent Haloperidol results in alterations of QKI expression levels. Furthermore, this change was observed using a concentration similar to plasma levels detected in patients, suggesting the possibility that Haloperidol affects astrocyte function.

The observed increase of QKI-7 mRNA expression is in agreement with previous findings by Aberg et al., showing increased gene expression of QKI in schizophrenic patients treated with typical antipsychotic agents [1,2]. A caveat to the interpretation to our results is that it is difficult to compare a transient increase of QKI-7 in astrocytoma cells with effects *in vivo *after chronic treatment of patients with Haloperidol and other drugs. However, our results indicate for the first time a link between Haloperidol action and QKI control within astrocytoma cells. These observations suggest a direct link between Haloperidol and QKI control in astrocytes, and they clearly indicate that the molecular pathway in which QKI is involved, is affected during haloperidol action both in *in vivo *and *in vitro *experiments.

Alteration of QKI expression after Haloperidol treatment has also been shown in mice [13], but the direction of the change was the opposite. The most reasonable explanation to the differences between the studies is that Naryan et al. worked with an *in vivo *model in mice whereas we worked with an *in vitro *model in humans. Differences in cell types between the species, as well as the absence of *in vitro *models of neuronal circuits and inter-cellular relationships present in the living brain makes the results difficult to compare. In other words, Naryan et al showed an effect of Haloperidol on QKI in mice neuronal networks while our study shows the individual effect of Haloperidol on glial cell lines. However, both studies coincide in a relationship between Haloperidol and QKI pathways. The molecular and cellular details of this interaction remain to be elucidated.

Previous studies found that dopamine receptors were expressed in primary astrocytic cultures [14]. We confirmed the expression of D2R in the U343 cells at the mRNA level. In addition, D1R and D2R proteins were previously detected in human astrocytoma cell lines, as published in the Human Protein Atlas . It is therefore possible that the increased expression of QKI-7 is mediated by Haloperidol binding to D2R. However, drug action through binding to other receptors cannot be excluded at this point. Future binding studies will clarify this point. If antipsychotic drugs affect QKI expression via binding to dopamine receptors, it would be expected that all drugs used in this study would affect QKI levels. However, this was not the case and the most pronounced effect was observed with Haloperidol. This does not exclude the possibility of D2R involvement since it is possible that Haloperidol may occupy more D2R than other agents when low doses are used.

Interestingly, when HOG cells were treated with Risperidone we observed a slight increase of QKI-6 mRNA levels. In U343 cells we did not detect an effect on QKI-6. Instead, we found a decrease of QKI-7 mRNA expression. Risperidone appears to have different effect on QKI in HOG cells and astrocytic cells, although the change in gene expression was not as pronounced as when Haloperidol was used.

## Conclusion

In summary, our results suggest a direct effect of Haloperidol treatment on QKI mRNA expression in astrocytes. A smaller effect on QKI expression was also detected when the cells were treated with Risperidone. The physiological effect of these changes on the symptoms of the patients, and the normal function of QKI in astrocytes and oligodendrocytes remain to be investigated. This knowledge could contribute to understand the molecular mechanism of action of antipsychotic drugs after binding to receptors, and it may also help to find new and better drug targets for the disease.

## Methods

### Treatment of cell lines

U343 and HOG cells were cultivated in Dulbecco's Modified Eagle Medium (DMEM, 61965-026, GIBCO™) supplemented with 10% fetal bovine serum (FBS, 10270-106, GIBCO™) and 1% penicillin-streptomycin solution (PEST, 15140-122, GIBCO™). Although some of the characteristics typical for oligodendrocytes and astrocytes may be changed in the glioma cells they are used for these experiments since it is not possible today to derive these cell types from a living human brain to perform this experiment. The cells were cultivated to about 30% confluence before treatment with antipsychotic drugs. Medium to treat the cells contained 1 μM Aripiprazole (A771000, Toronto Research Chemicals Inc.), 2 μM Clozapine (C6305, Sigma-Aldrich), 0.2 μM Haloperidol (H1512, Sigma-Aldrich), 0.25 μM Olanzapine (O253750, Toronto Research Chemicals Inc.) or 0.15 μM Risperidone (R525000, Toronto Research Chemicals Inc.), respectively, in DMEM supplemented with 10% FBS and 1% PEST. The concentrations used were in the typical range of blood plasma levels observed in patients treated with the drugs [9-12]. Three biological replicates were done for each test. The cells were treated for 6 or 24 hours. Control cells that were not treated, were incubated with the same medium without the antipsychotic agents, for 6 or 24 h. The cells were harvested by adding Trizol (Life Technologies, Sweden) directly to the cell plate after removing the medium, and the samples were collected in eppendorf tubes prior to RNA isolation.

In the initial experiment, we detected an increase of QKI-7 mRNA expression in Haloperidol treated U343 cells. To test whether the effect was dose dependent we performed an additional treatment with this drug using 10 times higher concentration (2 μM).

### RNA isolation and Gene expression analysis

RNA was isolated according to standard Trizol extraction procedures [15]. The concentration of RNA was measured with a NanoDrop^® ^ND-1000 Spectrophotometer (NanoDrop Technologies). RNA samples were stored at -70°C prior to use.

Reverse transcription reactions included 1 μL RT-buffer (10×), 2.2 μL MgCl_2 _(25 mM), 2 μL dNTPs (10 mM), 0.5 μL oligo dT (10 μg/L), 0.2 μL RNase inhibitor, 0.25 μL reverse transcriptase (Taqman Reverse Transcription Reagents, N808-0234, Applied Biosystem) and 3.85 μL of sample (about 500 ng of RNA). These reactions were incubated at 25°C for 10 min and 48°C for 1 hour.

Real-time RT-PCR was performed with an ABI PRISM 7000 Sequence Detection System (Applied Biosystems, Foster City, USA) as follows: 2 minutes at 50°C and 10 minutes at 95°C followed by 40 cycles of 15 seconds at 95°C and 1 minute at 60°C. Each reaction was carried out in a total volume of 25 μL, consisting of 9.8 μL Power SYBR^® ^PCR Master mix (Applied Biosystems, Foster City, USA), 0.3 μM of each primer (Thermo Electron Cooperation, Germany) and ~10–100 ng of cDNA. The primer for the reference gene (actin-β, ACTB) as well as for the QKI splice variants, QKI-5, QKI-6, QKI-7, were uniquely designed using Primer Express (Applied Biosystems, Foster City, USA), as described previously [2] (see Additional file 2 for primer sequences). The expression data was collected and analyzed with the ABI PRISM 7000 SDS software (Applied Biosystems, Foster City, USA).

### Statistical analysis

Messenger RNA expression levels of the QKI splice variants were normalized with the expression levels of the endogenous control ACTB. In other words, our target variable for the statistical analysis was the difference between QKI and ACTB expression on a logarithmic scale. To test the effect of antipsychotic treatment on QKI mRNA expression, we analyzed each cell line separately with a two-way ANOVA model, which included the factors treatment, time and the interaction between treatment and time. Comparisons between treated cells and their corresponding controls were carried out with linear contrasts, (i.e. with pre-planned t-test utilizing the pooled estimate of error variance). The statistical analysis was carried out in Proc GLM (SAS/STAT software, version 9.1.3, SAS institute Inc., Cary, NC).

## Abbreviations

ACTB: actin beta; D2R: dopamine 2 receptor; D3R: dopamine 3 receptor; FBS: fetal bovine serum; HOG: human oligodendrogial cell line; PEST: penicillin-streptomycin solution; PCR: polymerase chain reaction; QKI: *The quaking homolog, KH domain RNA binding (mouse)*; U343: astrocytoma cell line.

## Competing interests

The authors declare that they have no competing interests.

## Authors' contributions

LJ carried out cell work and real-time PCR experiments. PS participated in the statistical calculations. EJ participated in the study design and ELC performed cell work, expression studies, was involved in the statistical work and in the study design.

## Supplementary Material

Additional file 1 **Changes of QKI mRNA expression in U343 and HOG cells after treatment with different antipsychotic drugs.** The two upper tables show fold-change of QKI expression, standard deviation and p-value for each treatment in U343 and HOG cells. The lower table shows fold-changes of QKI expression, standard deviations and p-values after Haloperidol treatment, with the higher concentration (2 μM), of U343 cells. Changes with p-values < 0.05 are indicated with an asterisk. Two-fold differences are indicated with a box. Click here for file

Additional file 2 **Primer sequences used for detecting gene expression for ACTB, QKI-5, QKI-6 and QKI-7.** Primer sequences used to amplify ACTB-, QKI-5-, QKI-6-, and QKI-7 mRNA. Click here for file
