# Supplementary material for: Haloperidol changes mRNA expression of a QKI splice variant in human astrocytoma cells
Source: BMC Pharmacol. 2009 Mar 31;9:6. doi: 10.1186/1471-2210-9-6 (PMC2676266; doi:10.1186/1471-2210-9-6)
Supplement: Additional file 1 — Changes of QKI mRNA expression in U343 and HOG cells after treatment with different antipsychotic drugs. The two upper tables show fold-change of QKI expression, standard deviation and p-value for each treatment in U343 and HOG cells. The lower table shows fold-changes of QKI expression, standard deviations and p-values after Haloperidol treatment, with the higher concentration (2 μM), of U343 cells. Changes with p-values < 0.05 are indicated with an asterisk. Two-fold differences are indicated with a box. [file 1471-2210-9-6-S1.pdf]

Table 1a

		U343								
		QKI-5			QKI-6			QKI-7		
Time		Fold change	Stdev	p-value	Fold change	Stdev	p-value	Fold change	Stdev	p-value
6 hr	Arip	1.1	0.19	0.66	0.8	0.12	0.45	0.7	0.10	0.11
	Cloz	1.0	0.30	0.92	0.9	0.26	0.71	1.7	0.56	0.12
	Halop	1.1	0.13	0.68	1.0	0.05	0.96	**2.1**	**0.40**	**0.02***
	Olanz	0.9	0.38	0.53	1.0	0.59	0.75	0.8	0.24	0.25
	Risp	1.0	0.25	0.99	0.7	0.17	0.16	0.6	0.05	0.01 *
24 hr	Arip	1.1	0.37	0.85	1.1	0.12	0.61	1.1	0.10	0.56
	Cloz	0.7	0.35	0.09	0.9	0.65	0.32	0.6	0.45	0.02 *
	Halop	0.9	0.16	0.69	0.9	0.03	0.59	1.0	0.12	0.95
	Olanz	1.1	0.20	0.67	1.2	0.12	0.57	1.3	0.46	0.27
	Risp	1.0	0.04	0.99	1.1	0.23	0.62	1.1	0.16	0.58

Table 1b

		HOG								
		QKI-5			QKI-6			QKI-7		
Time		Fold change	Stdev	p-value	Fold change	Stdev	p-value	Fold change	Stdev	p-value
6 hr	Arip	1.1	0.15	0.54	0.9	0.43	0.59	0.7	0.18	0.11
	Cloz	1.1	0.20	0.43	1.0	0.28	0.98	1.0	0.11	0.62
	Halop	0.8	0.17	0.45	0.8	0.27	0.25	0.9	0.23	0.65
	Olanz	1.0	0.20	0.91	1.2	0.17	0.43	1.2	0.41	0.38
	Risp	1.0	0.05	0.70	1.7	0.37	0.03 *	1.4	0.37	0.09
24 hr	Arip	1.1	0.17	0.50	1.2	0.16	0.44	1.0	0.07	0.90
	Cloz	0.7	0.30	0.06	0.8	0.31	0.23	1.0	0.13	0.81
	Halop	1.1	0.14	0.49	1.2	0.34	0.50	0.8	0.19	0.18
	Olanz	0.9	0.18	0.58	1.1	0.36	0.59	0.8	0.13	0.25
	Risp	1.0	0.13	0.87	0.8	0.18	0.38	0.8	0.06	0.28

Table 1c

		QKI-7 in U343		
Time		Fold change	Stdev	p-value
6 hr	Halop	**3.1**	**0.32**	**< 0.0001** *
24 hr	Halop	**5.7**	**1.28**	**< 0.0001** *
