# Supplementary material for: Haloperidol changes mRNA expression of a QKI splice variant in human astrocytoma cells
Source: BMC Pharmacol. 2009 Mar 31;9:6. doi: 10.1186/1471-2210-9-6 (PMC2676266; doi:10.1186/1471-2210-9-6)
Supplement: Additional file 2 — Primer sequences used for detecting gene expression for ACTB, QKI-5, QKI-6 and QKI-7. Primer sequences used to amplify ACTB-, QKI-5-, QKI-6-, and QKI-7 mRNA. [file 1471-2210-9-6-S2.pdf]

Table 2

Gene	Forward	Reverse
ACTB	5'-gag cta cga gct gcc tga cg	5'-gta gtt tcg tgg atg cca cag gac t
QKI5	5'-aat cct tga gta tcc tat tga acc tag t	5'-gca tat cgt gcc ttc gaa ctt t
QKI6	5'-agc tac atc aat cct tga gta tcc tat tg	5'-tag cct ttc gtt ggg aaa gc
QKI7	5'-gct aca tca atc ctt gag tat cct att g	5'-cag gca tga ctg gca ttt ca
